# Microstructural landscape of amorphous carbon

**DOI:** 10.1093/nsr/nwae125

**Published:** 2024-03-28

**Authors:** Huiyang Gou

**Affiliations:** Center for High Pressure Science and Technology Advanced Research (HPSTAR), China

Due to its diverse bonding hybridization forms (sp, sp^2^ and sp^3^), amorphous carbon exhibits multifaceted properties and important application prospects, which have aroused extensive research interest [1–5]. In amorphous solids, some degree of atomic short- to medium-range order is thought to be ubiquitous, and the recent synthesis of new amorphous carbon has further increased our understanding of the atomic disorder in materials [4]. Shang *et al*. and Tang *et al*. reported different results for atomically disordered carbon [2,5], where the short-/medium-range order was demonstrated by the crystal-plane-like stripes or paracrystals in high-resolution transmission electron microscopy images, respectively. With reports of various complex atomic-scale structures, a question arises: does amorphous carbon exhibit intrinsic properties hidden by structural disorder?

Recently, a team led by Prof. Heng-An Wu at the University of Science and Technology of China published robust work on reacquainting atomic disorder in amorphous carbons [6]. They relied on the environment-dependent interaction potential (EDIP) developed by Prof. Marks [7] to achieve a phase diagram of amorphous carbons. Through microstructural topological analysis, the amorphous carbon was divided into six representative phases (Fig. 1a), namely, a disordered graphene network (DGN), high-density amorphous carbon (HDAC), amorphous diaphite (a-DG), amorphous diamond (a-D), paracrystalline diamond (p-D) and nano-polycrystalline diamond (NPD). DGNs have well-connected 3D-disordered graphene nanosheets, while HDACs exhibit only nanoscale graphene fragments with many heterocyclic defects. A-DG shows the hybrid characteristics of amorphous graphite and atomically disordered diamond [8]. Three kinds of atomically disordered diamonds (a-D, p-D and NPD) possess different degrees of atomic crystallization, where the presence of paracrystals endows p-D with a medium-range order [5].

**Figure 1.**
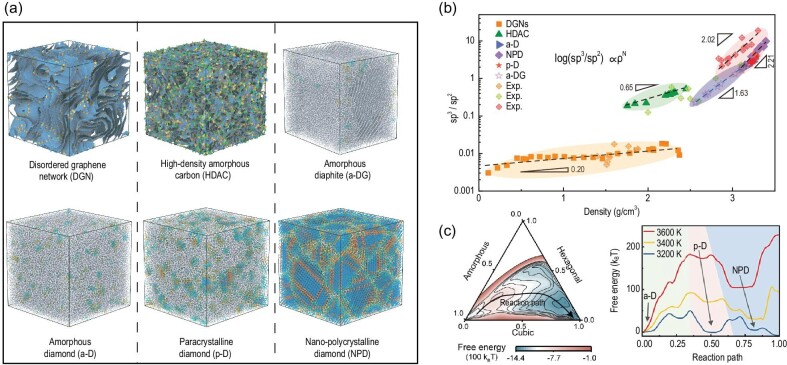
(a) Six representative phases of simulated amorphous carbons. (b) Phase diagram of amorphous carbons. (c) Free energy landscape of the nucleation of p-D and the temperature effect on the p-D nucleation process.

The phase diagram is plotted in the sp^3^/sp^2^ versus density plane (Fig. 1b), where the counterintuitive discontinuity arises from the inherent differences in the topology of the microstructure [6]. The dashed lines in Fig. 1b give the fitted power law, log(sp^3^/sp^2^) ∝ ρ^n^, for different amorphous carbons, where the index ‘n’ indicates that the microstructural stability of amorphous carbon can be regulated by changing sp^3^/sp^2^ under appropriate pressure-temperature conditions during phase transformation [6]. The proposed phase diagram can guide the discovery of transformations between different phases. Recent experiments have demonstrated that the synthesis of a-D and p-D [2,5] can be explained by the overlap of density between 3.1 and 3.4 g/cc, where a-D, p-D and NPD coexist in experiments. By constructing a free-energy landscape through metadynamics (Fig. 1c), they found that p-D was preferred over a narrow temperature range [9], while the inappropriate temperature led to a-D or NPD. These findings explain the distinct difference in the formation of p-D from a-D and NPD [2,5] and provide theoretical support for the experimental preparation of different tetrahedral amorphous carbons.

This timely discontinuous phase diagram presents a relatively comprehensive microstructural landscape for amorphous carbons with a wide range of densities, and its insights can help us better understand the various synthetic experimental equivalents [1–5,8]. The clear classifications and the power law revealed by the phase diagram allow us to rethink the underlying physics of the disorder of amorphous carbon and whether there is a potential pathway for synthesizing large-sized amorphous carbon through controlled phase transitions. This has significant implications for many important intrinsic properties and relationships hidden in diverse non-crystalline carbons that have yet to be discovered.

## References

[1] Zhang SS, Li ZH, Luo K et al. Natl Sci Rev 2022; 9: nwab140. 10.1093/nsr/nwab140 35070330 PMC8776544

[2] Shang Y, Liu Z, Dong J et al. Nature 2021; 599: 599–604. 10.1038/s41586-021-03882-9 34819685

[3] Zhu YB, Wang YC, Wu B et al. Nano Lett 2021; 21: 8401–8. 10.1021/acs.nanolett.1c02985 34591476

[4] San-Miguel A . Nature 2021; 599: 563–4. 10.1038/d41586-021-02957-x 34819687

[5] Tang H, Yuan X, Cheng Y et al. Nature 2021; 599: 605–10. 10.1038/s41586-021-04122-w 34819683

[6] Zhu Y, Fang Z, Zhang Z et al. Natl Sci Rev 2024; 11: nwae051. 10.1093/nsr/nwae051 38504723 PMC10950053

[7] Marks NA . Phys Rev B 2000; 63: 035401. 10.1103/PhysRevB.63.035401

[8] Li Z, Wang Y, Ma M et al. Nat Mater 2023; 22: 42–9. 10.1038/s41563-022-01425-9 36522415 PMC9812777

[9] Zhang ZT, Fang ZY, Wu HA et al. Nano Lett 2024; 24: 312–8. 10.1021/acs.nanolett.3c04037 38134308

